# Complete Genome Sequence of Rhodopseudomonas palustris RCB100, an Anoxygenic Phototroph That Degrades 3-Chlorobenzoate

**DOI:** 10.1128/MRA.00043-21

**Published:** 2021-04-15

**Authors:** Irshad UI Haq, Kathryn R. Fixen

**Affiliations:** a Department of Plant and Microbial Biology, College of Biological Sciences, University of Minnesota, St. Paul, Minnesota, USA; University of Delaware

## Abstract

The purple nonsulfur bacterium Rhodopseudomonas palustris RCB100 anaerobically degrades 3-chlorobenzoate (3-CBA), a halogenated pollutant. R. palustris RCB100 uses 3-CBA as a carbon source, while most R. palustris strains cannot. We report the complete genome sequence of strain RCB100 to help gain insight into how this bacterium degrades 3-CBA.

## ANNOUNCEMENT

Chlorobenzoic acids (CBAs) are environmental pollutants generated from the breakdown of chlorinated compounds like polychlorinated biphenyls (PCBs) (1–3). CBA-degrading bacterial species that have been isolated include the purple nonsulfur bacterium, Rhodopseudomonas palustris RCB100, which was isolated from Cascadilla Creek soil in Ithaca, NY (4–9). Degradation of 3-CBA by RCB100 involves the conversion of 3-CBA to 3-chlorobenzoyl-coenzyme A (CoA) and reductive dehalogenation to benzoyl-CoA, which is degraded by the benzoyl-CoA pathway (9). The ability of RCB100 to metabolize 3-CBA may be due to a single nucleotide change in *aliA*, which encodes a coenzyme A ligase, but this single nucleotide polymorphism (SNP) alone is not sufficient to allow a strain unable to metabolize 3-CBA to degrade 3-CBA (10). This indicates that RCB100 encodes additional SNPs or genes required for 3-CBA degradation. We report the complete genome sequence of strain RCB100 to gain insight into how this strain can degrade 3-CBA.

Genomic DNA was isolated from 10 ml of liquid culture grown in minimal mineral medium (11) containing 20 mM acetate and 0.1% yeast extract and incubated anaerobically in the light. Genomic DNA was extracted using the Qiagen Puregene yeast/bacteria kit B. The Microbial Genome Sequencing Center (MiGS; https://www.migscenter.com/) did the library preparation and sequencing. For preparation of the Illumina library, MiGS used Nextera XT according to the workflow described in reference 12 and performed the sequencing using the NextSeq 550 platform. The library for Nanopore was prepared using the SQK-LSK109 kit as per the manufacturer’s specification and sequenced on the Oxford Nanopore Technologies (ONT) MinION v9.4.1 flow cell. Illumina sequencing generated 2,376,223 reads (649,890,819 bp) with an average read length of 137 bp, and Nanopore sequencing resulted in 100,324 reads (676,353,988 bp) with an average read length of 6,742 bp. All tools were run with default parameters unless otherwise specified. Guppy v4.2.2 (Oxford Nanopore Technologies) was used for base calling. For Illumina reads, bcl2fastq v2.20 (https://support.illumina.com/sequencing/sequencing_software/bcl2fastq-conversion-software/documentation.html) was used to trim the adapters and for quality control. Porechop (https://github.com/rrwick/Porechop) was used for the ONT-generated sequencing reads. Hybrid assembly with all reads was performed with Unicycler v0.4.8 (13). QUAST (14) was used to determine the assembly statistics. Two contigs were generated with an *N*_50_ value of 5,448,862 bp. Assembly annotation was performed with Prokka v1.14.6 (15), using the following parameters: *prokka assembly.fasta*; *kingdom Bacteria*; *genus Rhodopseudomonas*; *species palustris*; *strain RCB100*; *gcode 11*; *compliant*; *outdir PRJNA683609*; *locustag I8G32*; and *prefix I8G32-RCB100*. The resulting assembled genome is 5,457,289 bp with a GC content of 65.03% and consists of a single circular chromosome (5,448,862 bp) and a circular plasmid (8,427 bp). To identify SNPs present in the genome of RCB100 compared to the lab strain CGA009, snippy v4.6.0 (https://github.com/tseemann/snippy) was used (parameters, *snippy*; *reference CGA009.gbff*; *ctgs assembly.fasta*; *ram 2*; *cpus 4*; *outdir CGA009_snps*) and identified 37 variants, including the SNP in *aliA* reported by Samanta and Harwood (10).

### Data availability.

The BioProject and BioSample accession numbers are PRJNA683609 and SAMN17028957, respectively. The GenBank accession numbers for the assembled genome are CP066699 for the chromosome and CP066700 for the plasmid sequence. The raw sequencing reads are available in the Sequence Read Archive (SRA) under the accession numbers SRR13238779 and SRR13238693.
